# The interplay between AR, EGF receptor and MMP-9 signaling pathways in invasive prostate cancer

**DOI:** 10.1186/s10020-018-0035-4

**Published:** 2018-06-27

**Authors:** Anna Mandel, Per Larsson, Martuza Sarwar, Julius Semenas, Azharuddin Sajid Syed Khaja, Jenny L. Persson

**Affiliations:** 1 Department of Molecular Biology, Umeå University, 901 87 Umeå, Sweden; 2 Division of Experimental Cancer Research, Department of Translational Medicine, Clinical Research Centre, Lund University, Jan Waldenströms gatan 35, 205 02 Malmö, Sweden

**Keywords:** Prostate cancer, Cancer metastasis, Epidermal growth factor receptor, Androgen receptor and androgen

## Abstract

**Background:**

Metastatic Prostate cancer (PCa) cells have gained survival and invasive advantages. Epidermal growth factor (EGF) receptor is a receptor tyrosine kinase, which may mediate signalling to promote progression and invasion of various cancers. In this study, we uncovered the molecular mechanisms underlying the interconnection among the androgen receptor (AR), matrix metalloproteinase-9 (MMP9) and EGFR in promoting PCa progression.

**Methods:**

Immunohistochemical analysis of the tissue microarrays consisting of primary and metastatic PCa tissues was performed. The clinical importance of EGFR and its association with survivals were analyzed using three cohorts from MSKCC Prostate Oncogenome Project dataset (For primary tumors, *n* = 181; for metastatic tumors *n* = 37) and The Cancer Genome Atlas Prostate Adenocarcinoma Provisional dataset (*n* = 495). Targeted overexpression or inhibition of the proteins of interests was introduced into PCa cell lines. Treatment of PCa cell lines with the compounds was conducted. Immunoblot analysis was performed.

**Results:**

We showed that AR, MMP-9 and EGFR are interconnect factors, which may cooperatively promote PCa progression. Altered EGFR expression was associated with poor disease-free survival in PCa patients. Induced overexpression of AR led to an increase in the expression of EGFR, p-GSK-3β and decrease in p27 expression in PCa cell lines in the presence of androgen stimulation. Overexpression of MMP9 significantly induced EGFR expression in PCa cells. Inhibition of PIP5K1α, a lipid kinase that acts upstream of PI3K/AKT greatly reduced expressions of AR, MMP-9 and EGFR.

**Conclusions:**

Our findings also suggest that PCa cells may utilize AR, EGFR and MMP-9 pathways in androgen-dependent as well as in castration-resistant conditions. Our data suggest a new therapeutic potential to block cancer metastasis by targeting AR, EGFR and MMP-9 pathways in subsets of PCa patients.

## Background

The derivative of the androgen testosterone, dihydrotestosterone (DHT) is the most abundant sex-hormone within the prostate and has a high binding affinity to androgen receptor (AR) (Feldman and Feldman 2001). Prostate cancer (PCa) cells in the initial stages of tumour development are responsive to androgens, however cancer cells often progress to a hormone-refractory state, termed castration-resistant prostate cancer (CRPC) (Denmeade and Isaacs 2002). AR is a transcription factor that regulates a panel of genes controlling the growth of prostate cells. Increased AR expression has been shown to affect the activation of its target genes, thereby promoting proliferation of PCa cells and rendering PCa resistant to androgen deprivation therapy (Hsu et al. 2005; Wang et al. 2005). Elevated level of AR expression is also associated with CRPC metastasis (Grasso et al. 2012; Shen and Abate-Shen 2010). This suggests that overexpression of AR originating from amplification or enhanced phosphorylation may allow PCa cells to circumvent androgen-dependent signaling.

One of the major features of PCa is its heterogeneity. PCa often contains a mixture of heterogeneous populations including cancer cells, stromal cells, fibroblasts and tumor-specific extracellular matrix (ECM) (Joyce and Pollard 2008; Kim et al. 2011). It has become clear that abundant growth factors are not only secreted by cancer cells, but are also produced by tumor-specific stromal cells, fibroblasts, ECM constituents and other cell types. The regulation of growth factors and their receptors is mediated through autocrine- or paracrine-dependent manners (Blume-Jensen and Hunter 2001; Bruzzese et al. 2014; Lemmon and Schlessinger 2010). In PCa, abnormal levels of growth factors are frequently observed in serums and in tumor tissues obtained from PCa patients (Reynolds and Kyprianou 2006). Remarkably, growth factors produced by the bone matrix and bone marrow niche promote growth and proliferation of metastasized PCa cells (Gleave et al. 1991; Kimura et al. 2010). Epidermal growth factor (EGF) family of growth factors interact with their receptors including EGF receptor (also known as ErB-1 or Her 1), Her 2/neu (ErbB-2), Her 3 (ErbB-3) and Her 4 (ErbB-4) (Casaletto and McClatchey 2012). Upon binding to its ligands, EGFR becomes active by formation of homodimers. The homodimers of EGFR phosphorylate and interact with corresponding downstream factors, which regulate fundamental cellular events including proliferation, survival and migration (Chong and Jänne 2013; Wells 1999). Alternatively, EGFR can be activated via hetero-dimerization with other receptors belonging to the epidermal growth factor receptor family of tyrosine kinases (Ono and Kuwano 2006). Similarly to that of their ligands, alterations in the expression and activity of EGFR also occur in PCa (De Miguel et al. 1999). Expression of EGFR is low in normal prostate tissues (Traish and Wotiz 1987), while it is highly expressed in primary and metastatic PCa tissues (Di Lorenzo et al. 2002; Hofer et al. 1991). Furthermore, EGFR and HER-2 have been revealed to play a significant role in metastasis to the bone marrow (Day et al. 2017; Lu and Kang 2010), and these factors exhibited elevated activity in tumour initiating cells (TICs) and circulating tumour cells (CTCs) (Day et al. 2017). Taken together these data suggest a role of EGFR in the development and progression of PCa. Since excess levels of EGF and EGFR are produced by both PCa cells and tumor-specific stromal/fibroblasts, it is likely that EGFR signalling in cancer cells is activated via the production of binding ligands by both cancer cells and tumor-specific stromal/fibroblasts through paracrine and autocrine loops, leading to the growth and survival of PCa cells in the absence of androgens (Di Lorenzo et al. 2002; Traish and Wotiz 1987).

EGFR and its ligands may replace androgens to enhance phosphorylation of AR or act as AR co-regulators to promote activation of its downstream genes. It has been proposed that forced overexpression of HER2 kinase increases AR expression and promotes growth of hormone-refractory PCa cells through AR signaling (Craft et al. 1999; Yeh et al. 1999). Dual repression of EGFR and HER-2 has been shown to impair PCa tumour cell proliferation and survival (Chen et al. 2011; Day et al. 2017). Further, EGFR/ERBB2 kinase activity was revealed to be significantly up-regulated in LNCaP cells co-cultured with osteoblastic cells as determined by multiplex kinase activity profiling. This study hints that EGFR activity is stimulated by tumor-associated bone cells (Bratland et al. 2009). Activation of EGFR is also mediated by type 1 insulin-like growth factor (IGF) and extracellular matrixes, which are produced by the tumor-associated microenvironment during PCa metastases in the bone marrow (Chott et al. 1999). However, the role of EGFR in metastases and the precise mechanisms underlying EGFR activation by the tumor-associated microenvironment are largely unknown.

Matrix metalloproteinase-9 (MMP-9) is involved in degradation of ECM and vascular remodeling during tumor cell invasion (Heissig et al. 2002). It has been shown that MMP-9 produced by fibroblasts promotes mitogenic induction in breast cancer cells by enhancing endothelial cell survival and function in an in vitro co-culture model (Shekhar et al. 2001). MMP-9 may amplify local angiogenesis due to its ability to cleave membrane-bound vascular endothelial growth factor (VEGF), hence elevating the level of functional VEGF in tumors (Bergers et al. 2000). Due to the role of MMP-9 in cancer metastasis, the association between EGFR and MMP-9 is an intriguing target for the investigation of EGFR’s involvement in PCa invasion.

During the past years, several new classes of inhibitors against EGFR have been developed and have shown promising effects in targeting metastasized cancers of the lung, breast, colorectal system, and head and neck (Bertotti et al. 2015; Blaszczak et al. 2017; Chong and Jänne 2013; Munagala et al. 2011). The EGFR inhibitors cetuximab, panitumumab and geftinib have been approved by FDA and are currently used for treatment of patients with lung cancer, and head and neck cancers (Blaszczak et al. 2017; Chong and Jänne 2013; Kazandjian et al. 2016). These inhibitors induce apoptosis in cancer cells by blocking multiple EGFR-dependent growth and survival signaling pathways (Chong and Jänne 2013). Third-generation EGFR inhibitors such as rociletinib have been approved for treatment of EGFR-mutated non–small-cell lung cancer (Chabon et al. 2016; Eberlein et al. 2015; Piotrowska et al. 2015). The effects of EGFR inhibitors on CRPC remain to be further investigated in preclinical models and in patient-based clinical trials. A Phase II study in CRPC of lapatinib, an inhibitor of EGFR and human epidermal growth factor receptor 2 (HER2), showed prostate-specific antigen (PSA) response only in a very small number of patients (Whang et al. 2013). Dual inhibition of EGFR and HER2 poses as a promising prospect in terms of PCa therapy (Ahmad et al. 2011; Chen et al. 2011; Day et al. 2017; Sridhar et al. 2010), however to date, trials have been unsuccessful. It is of importance to gain deeper understanding of the cellular mechanisms underlying the interplay between PCa cells and PCa-associated microenvironment during progression of CRPC, and specifically to gain deeper knowledge about the role of EGFR in proliferation, survival and migration of PCa cells and PCa-associated cells during development of CRPC.

The aim of our study was to investigate the mechanisms underlying the interplay between AR and EGFR as well as MMP-9 and EGFR in PCa progression. We found that androgen treatment of both control and AR-overexpressing PCa cells led to a significant increase in the activation of EGFR and its associated activity with PI3K/AKT pathways, thus presumably allowing PCa cells to gain survival and invasive advantages. We also showed that EGFR is likely to be involved in PCa invasive mechanisms via MMP-9 signaling. Our study provides information on clinical and molecular bases suggesting that AR and EGFR are elements of interlinked signalling pathways, which allow PCa cells to use alternative mechanism without consuming large quantities of androgens, thereby bypass androgen-dependent pathways.

## Methods

### Tissue specimens, tissue microarrays and mRNA expression data

Tissue microarrays (TMAs) containing primary (*n* = 17) and metastatic PCa lesions (*n* = 43) from 14 PCa patients were constructed at Department of Clinical Pathology and Cytology, Skåne University Hospital, Malmö. The tumor tissues were reviewed and selected by two pathologists specialized in urology. The selected tissue cores were collected, paraffin-embedded and sectioned for histological analysis as described (Voduc et al. 2008). For comparison of EGFR between normal prostate free of pathological conditions, primary tumors and metastatic lesions gene expression data from the dataset GDS2545 in the Gene Expression Omnibus (GEO) database at the National Center for Biotechnology Information (NCBI) website was used. The dataset was obtained by performing Affymetrix HG-U95Bv2 oligonucleotide array platform as described (Chandran et al. 2007; Yu et al. 2004). The mean mRNA values of genes of interests from a total 146 human samples in the dataset GDS2546 were used in the present study. The samples included normal prostate tissues adjacent to tumor (*N* = 58), primary tumor (*N* = 64), and the metastatic lesions (*N* = 24) from liver, para aortic lymph node, para-tracheal lymph node, retroperitoneal lymph node, lung and adrenal gland of 4 patients with CRPC. For mRNA expression and copy number alteration (CNA) data for EGFR, the disease-free survival (DFS) data was extracted from the open-access cBioPortal databases. MSKCC Prostate Oncogenome Project dataset (For primary tumors, *n* = 181; for metastatic tumors *n* = 37) and The Cancer Genome Atlas (TCGA) Prostate Adenocarcinoma Provisional dataset (For tumors taken from primary site *n* = 495) as described (Robinson et al. 2010; Taylor et al. 2010). The follow-up time from diagnosis to disease recurrence known as biochemical recurrence (BCR) ranged from 1 to 60 months was used for analysis of DFS. The study was approved by the Ethics Committee, Lund University, and the Helsinki Declaration of Human Rights was strictly observed.

### Immunohistochemistry analysis

Immunohistochemistry on TMAs was performed as previously described (Wegiel et al. 2005). The staining procedure was performed using a semiautomatic staining machine (Ventana ES, Ventana Inc., Tucson, AZ). For immunohistochemical analysis of xenograft mouse organs, tissues or tumors were fixed in 4% paraformaldehyde for 24 h and embedded in paraffin. For histology analysis, the sections were stained with hematoxylin-eosin (H&E) and were subjected to analysis using an Olympus BX51 microscopy. Immunostaining of tumor tissues using antibodies was performed as previously described (Wegiel et al. 2008). The sections were viewed under an Olympus BX51 microscope at magnification of 20× or 40×. The slides were scanned and viewed; microphotographs were taken by using a high resolution scanner (ScanscopeCS, Aperio, Vista, CA). The staining intensity was scored as 0 (negative), 1 (weakly positive or positive), 2 (moderate positive), 3 (strongly or very strongly positive) using an arbitrary semi-quantitative scale.

### Cell culturing and treatments

We used VCaP cells that is the “Vertebral-Cancer of the Prostate” cell line, which was established from prostate cancer tissue harvested from a metastatic lesion to a lumbar vertebral body of a patient with hormone refractory prostate cancer. The cells express AR and prostate-specific antigen (PSA). PC-3 cells is the castration-resistant prostate cancer cell line, which does not express AR and is insensitive to androgen stimulation. The cells were purchased from American Type Culture Collection (Manassas, VA, USA). Cells were maintained in RPMI-1640 medium or Ham’s F-12 medium supplemented with 10% fetal bovine serum (FBS), 1% penicillin-streptomycin-neomycin (PSN) and 2 mM L-Glutamine. For treatment, cells were grown for 24 h in phenol red-free RPMI-1640 medium containing 10% charcoal stripped-serum and were subsequently treated with agents for 24 h. Dihydrotestosterone (DHT) at a final concentration of 5 nM in 0.1% DMSO, or PIP5K1 alpha inhibitor, a diketopiperazine fused C-1 indol-3-yl substituted 1,2,3,4-tetrahydroisoquinoline derivative, ISA-2011B (Semenas et al. 2014) at a final concentration of 50 μM in 0.1% DMSO, or solvent DMSO 0.1% for 48 h was applied as treatment.

### Plasmids transfection

For transient transfection studies, pCMV-AR containing full-length AR and pCMV control vectors were kindly provided by Dr. Yvonne Giwercman at Department of Translational Medicine, Lund University, Sweden. pLX304 (Addgene, MA, USA); pLX304-MMP9 (PlasmID, Harvard Medical School, MA, USA) were used. For introduction of the plasmids, Lipofectamine® 2000/3000 transfection reagent (Life Technologies, Paisley, UK), TransIT-TKO® and TransIT-X2® (Mirus Bio, WI, USA) were used according to the manufacturer’s instructions.

### Immunoblot analysis and source of antibodies

The cells or tumor tissues were harvested and lysed in ice-cold RIPA buffer. Proteins (20–40 μg) were separated using 10 and 12% SDS-PAGE gels and transferred onto nitrocellulose membranes. Signals were visualized using the Enhanced ChemiLuminescence detection system (Pierce, Rockford, USA) and documented with an AlphaImager CCD system. Densitometric quantification of immunoblots was performed by the ImageJ Image Analysis Software (NIH, Baltimore, USA) and represented as fold change relative to control and were normalized relative tond GAPDH bands. The following primary antibodies were used in this study: Monoclonal antibodies against estrogen receptor (ER) alpha (Nordic BioSite, Taby, Sweden), MMP-9 (Abcam, Cambridge, UK), EGFR (Abcam, Cambridge, UK), p-GSK-3 beta, p27, AR, GAPDH (Santa Cruz Biotechnology Inc., Santa Cruz, CA). Secondary antibodies used: HRP-conjugated anti-mouse IgG, anti-rabbit IgG (GE Healthcare) and anti-goat (Santa Cruz Biotechnology Inc., Santa Cruz, CA).

### Statistical analysis

Student t-test was used for statistical analyses of the experimental data. Spearman rank correlation test was used to establish the level of correlation between mRNA expressions of relevant factors. Distribution of disease-free survival (DFS) was estimated by the method of Kaplan-Meier, with 95% confidence intervals. Differences between survival curves were calculated applying the log-rank test using the statistical program SPSS version 24.0. *P*-values equal to or less than 0.05 were considered to be statistically significant.

## Results

### Clinical importance of EGFR expression and its correlation with AR in primary and metastatic PCa tissues from patients

**Fig. 1:**
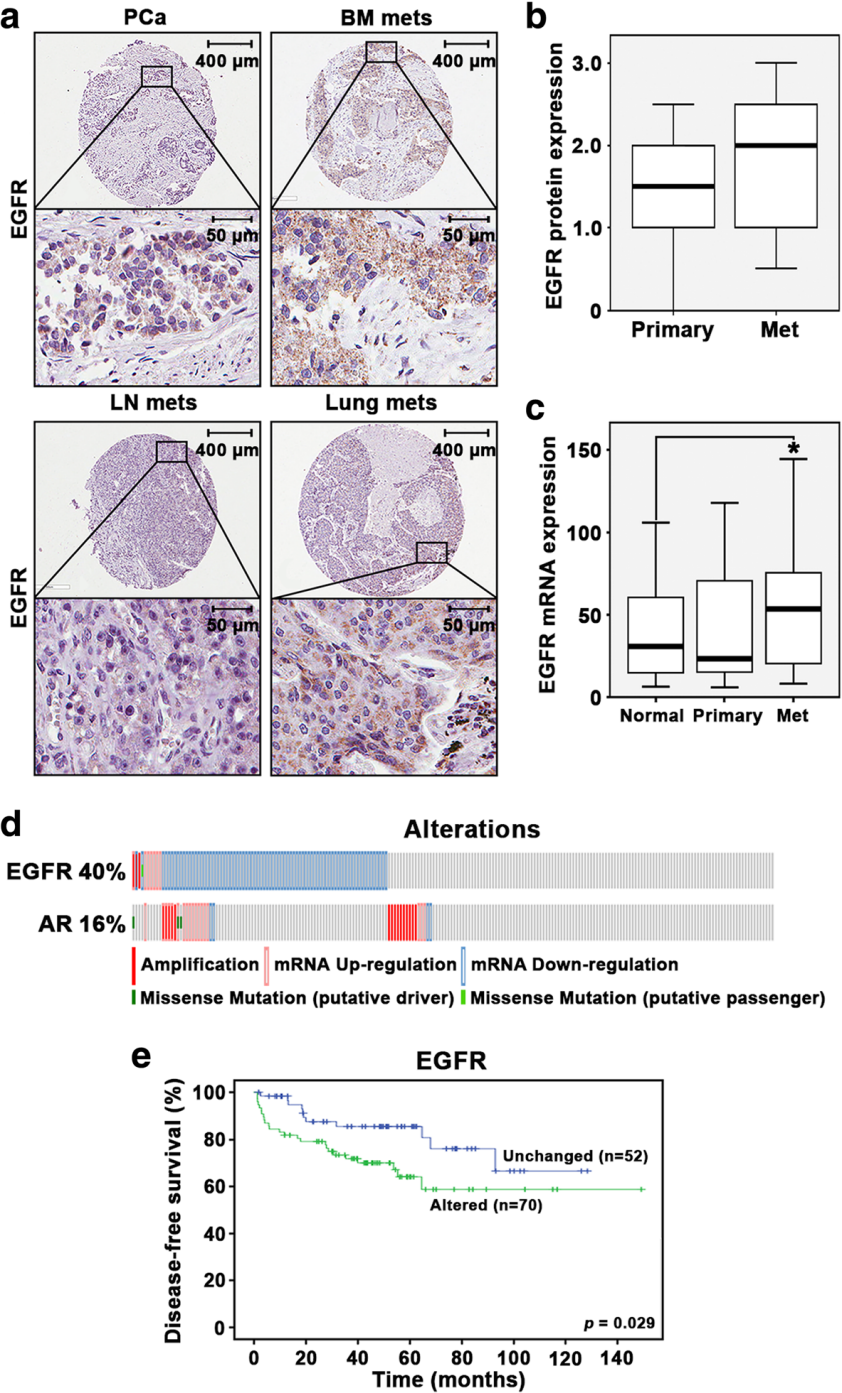
Evaluation of the clinical importance of EGFR and its correlation with AR in prostate cancer patients. **a** Immunohistochemical analysis of EGFR expression in primary PCa (*n* = 17), and in bone, lymph node and lung metastatic PCa sites (*n* = 43). The TMA staining intensity shows that EGFR protein expression is highest in bone metastatic PCa lesions. **b** Box plot showed the comparison in EGFR protein expression between primary PCa (*n* = 17) and metastatic lesions (*n* = 43) (*p* = 0.147). **c** Box plot showed the comparison in EGFR mRNA expression between normal prostate (*n* = 58), primary PCa (*n* = 64) and metastatic lesions (*n* = 24) (*p* = 0.05). **d** Gene and mRNA alteration profiles of EGFR and AR in PCa patients (*n* = 216) where 40% of patients (*n* = 86) exhibited EGFR alterations on the gene and mRNA level, while 16% of patients (*n* = 35) exhibited discrepancies in AR gene and mRNA expression. MSKCC Prostate Oncogenome Database was used. **e** Kaplan-Meier survival curve revealed that patients with alterations in EGFR (*n* = 70) suffered poorer disease-free survival (DFS) as compared to those without alterations (*n* = 52), and this difference was statistically significant (*p* = 0.029). MSKCC Prostate Oncogenome Database was used

To evaluate clinical importance of EGFR and its correlation with AR expression in PCa patients, we used TMAs consisting of primary PCa (*n* = 17), and PCa metastatic tissues (*n* = 43). The TMAs were immuno-stained with antibodies against EGFR. EGFR was expressed in primary and metastatic lesions including lymph nodes, lungs and bones with bone metastatic TMAs having the highest staining intensity against EGFR protein expression (Fig. 1a). There was a clear trend that EGFR protein expression was higher in metastatic PCa tissues than that in primary PCa tissues, although statistical significance was not achieved, probably due to the small sample size (*p* = 0.147) (Fig. 1b). Pearson correlation test revealed that there was a significantly positive correlation between AR and EGFR protein expression (r^2^ = 0.348, *p* = 0.011) in primary and metastatic PCa tissues from this patient cohort (Table 1). In order to further examine the clinical relevance of EGFR expression, we compared EGFR mRNA expression between normal prostate tissues adjacent to the prostate tumor tissues, primary PCa tissues, as well as PCa metastatic lesions. We found that EGFR expression was significantly higher in metastatic lesions compared with the normal prostate tissues (*p =* 0.05). There was a trend that EGFR expression was increased in metastatic lesions compared with primary prostate tumors, however, the statistical significance was not achieved (Fig. 1c). This data suggests that EGFR expression was elevated in metastatic PCa.

**Table 1:** Pearson’s correlation of protein expression between AR and EGFR

		EGFR
AR	Correlation coefficient	0.348*
	Significance (*p* value)	0.011

The analysis implies significant positive correlation between the two factors. The correlation between AR and EGFR is significant at the 0.05 level

(*p < 0.05)

We next examined EGFR mRNA expression in PCa tissues originating from the primary site (*n* = 495) using The Cancer Genome Atlas (TCGA) Prostate Adenocarcinoma Provisional database. Spearman correlation test revealed that there was a significantly positive correlation between AR and EGFR mRNA expression (r^2^ = 0.756, *p* < 0.001) in primary PCa tissues (n = 495) (Table 2). Alterations in EGFR gene were found in 40% of tumor tissues, alterations in AR gene were detected in 16% of tumors as assessed using the dataset from the MSKCC Prostate cBioportal Database (Fig. 1d). To examine whether alterations in EGFR might be associated with patient outcome, we performed Kaplan-Meier survival analysis. We observed that patients with alterations in EGFR (*n* = 70) suffered poorer DFS as compared to those without alterations (*n* = 52), and this difference was statistically significant (*p* = 0.03) (Fig. 1e). These data suggested that alterations in both AR and EGFR may be interlinked events and are associated with poor patient outcome in PCa.

**Table 2:** Spearman’s correlation of mRNA expression between AR and EGFR

		EGFR
AR	Correlation coefficient	0.756**
	Significance (*p* value)	0.000

The analysis implies significant positive correlation between the two factors. The correlation is significant at the 0.001 level

(**p < 0.001)

### The effect of elevated AR expression on EGFR and its associated signaling in VCaP cells

**Fig. 2:**
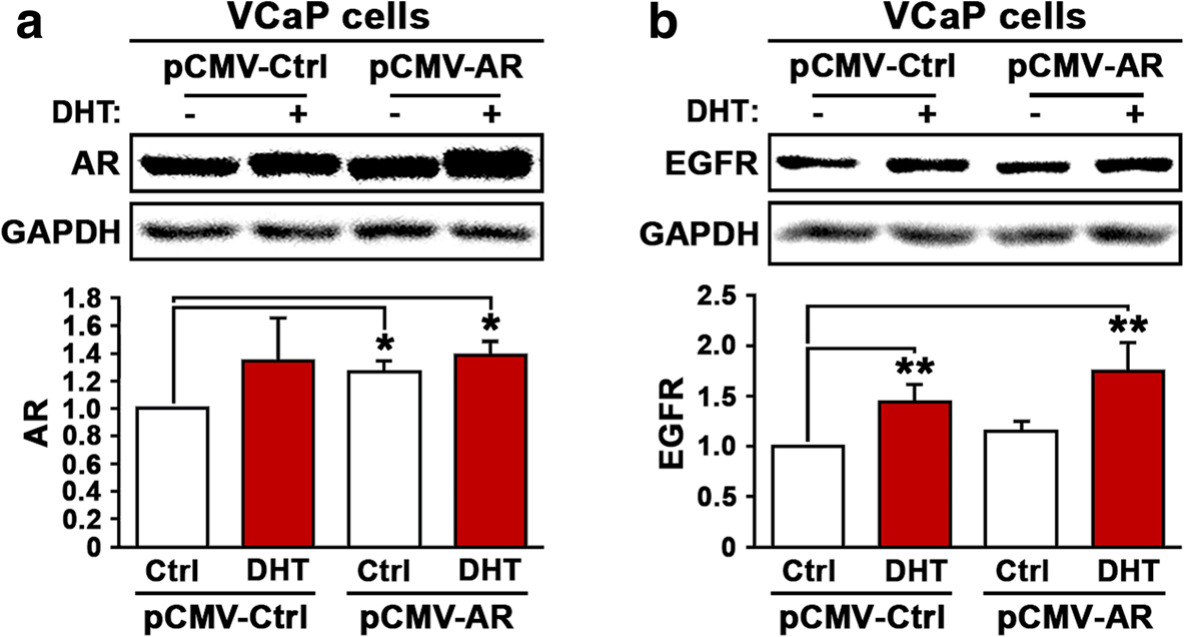
Evaluation the effect of overexpression of AR and DHT treatment on expression of EGFR in VCaP cells. **a** Immunoblot analysis was performed to examine the expression of AR in VCaP cells that were transfected with pCMV control vector (pCMV-Ctrl) or pCMV-AR vector (pCMVAR) and followed by treatment with DHT or vehicle control. **b** Expression of EGFR protein in VCaP cells that were transfected with pCMV control vector (pCMV-Ctrl) or pCMV-AR vector (pCMVAR) and followed by treatment with DHT or vehicle control. Antibody against GAPDH was used as loading control. Data presented is average of three independent experiments (±SD). *p* < 0.05 is indicated by “*”, *p* ≤ 0.01 is indicated by “**”

To examine whether AR signaling affects EGFR protein expression, we used VCaP cells derived from metastatic lesions of CRPC. We induced overexpression of AR by transfecting VCaP cells with pCMV-AR or pCMV control vectors. Immunoblot analysis confirmed the overexpression of AR in VCaP cells transfected with pCMV-AR vector compared with the cells transfected with pCMV control vector (*p* = 0.04) (Fig. 2a). To examine whether induction of androgen may further enhance AR expression in VCaP cells, we treated VCaP cells overexpressing AR or transfected with control vector with DHT at 5 nM dose. There was a trend that DHT treatment increased AR expression in VCaP cells expressing control vector, however, statistical significance was not achieved (Fig. 2a). DHT treatment enhanced AR expression in VCaP cells expressing the pCMV-AR vector and this was statistically significant (*p* = 0.03) (Fig. 2a). We next investigated whether elevated level of AR with or without the presence of its ligand androgen may have any effect on EGFR expression. We examined EGFR expression in VCaP cells expressing pCMV-AR or control vector in the presence of absence of 5 nM DHT. DHT stimulation significantly induced an upregulation of EGFR expression in VCaP cells expressing pCMV control vector as determined by immunoblot analysis (*p* = 0.01; Fig. 2b). Induced overexpression of AR alone had no effect on EGFR expression, however, DHT treatment of VCaP cells that overexpressed AR resulted in a dramatic increase in EGFR expression (*p* = 0.01; Fig. 2b). These data suggest that androgen and the ligand stimulation of AR by androgen have a significant positive effect on EGFR expression.

**Fig. 3:**
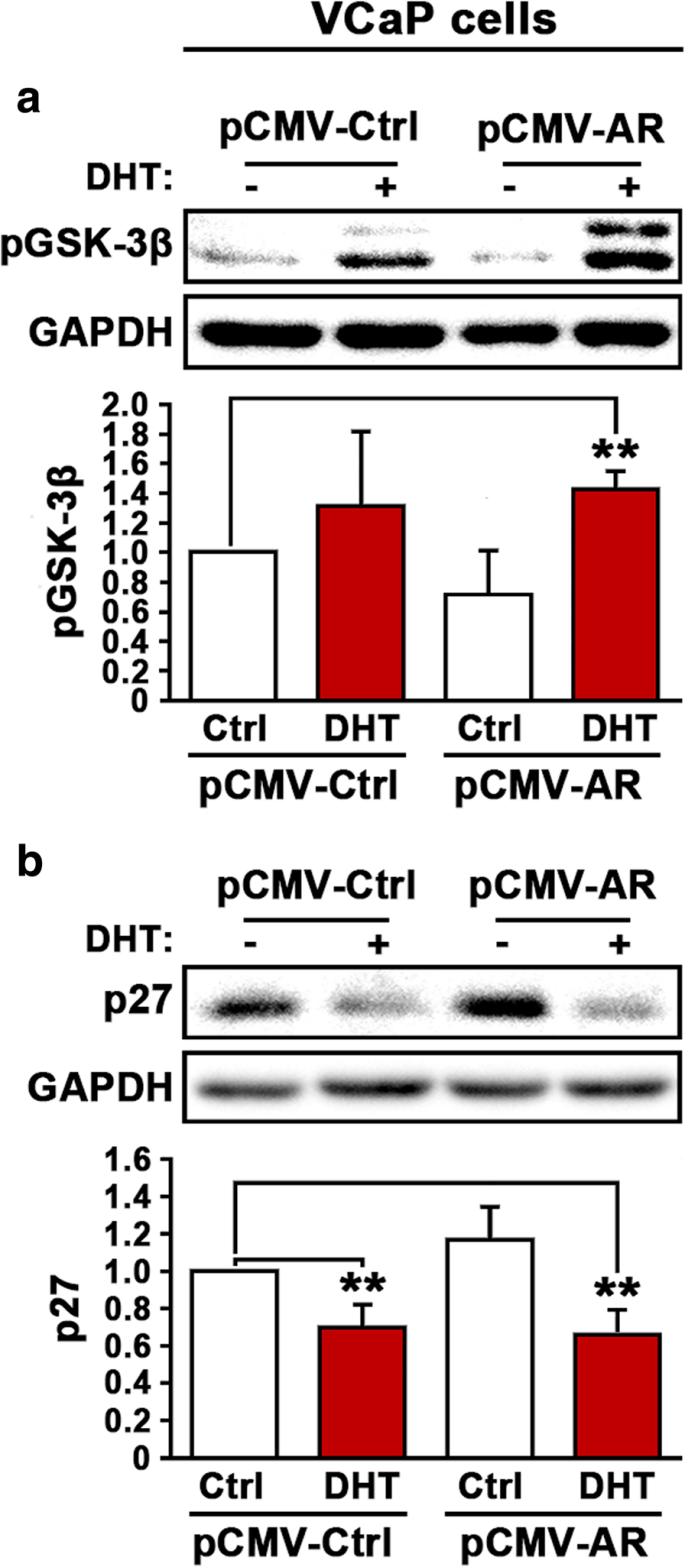
Evaluation the effect of overexpression of AR and DHT treatment on EGFR-related downstream effectors of AKT. **a** Immunoblot analysis was performed to examine the expression of p-GSK-3β in VCaP cells that were transfected with pCMV control or pCMV-AR vectors followed by treatment with DHT or vehicle control. **b**. Immunoblot analysis was performed to examine the expression of p27 in VCaP cells that were transfected with pCMV control or pCMV-AR vectors followed by treatment with DHT or vehicle control. Antibody against GAPDH was used as loading control. Data presented is average of three independent experiments (±SD). *p* < 0.05 is indicated by “*”, *p* ≤ 0.01 is indicated by “**”

Since PI3K/AKT axis acts as a mediator between EGFR and AR signaling, we examined the effects of DHT stimulation and AR overexpression on AKT down-stream factors, p-GSK-3β and p27. DHT treatment or AR overexpression alone had no significant effect on p-GSK-3β, however, DHT treatment and AR overexpression additively increased the expression of p-GSK-3β significantly in VCaP cells (*p* = 0.003; Fig. 3a). P27 is a key cell cycle inhibitor, and decreased level of p27 is associated with increased proliferation. We observed that DHT treatment resulted in decreased expression of p27 (*p* = 0.01; Fig. 3b). The combination of DHT treatment and AR overexpression also significantly reduced p27 expression in VCaP cells (*p* = 0.01; Fig. 3b). The findings suggest that there is a functional link between AR/androgen and EGFR and its associated cellular signaling in PCa cells.

### An association between AR and MMP-9 signaling, and EGFR protein expression in VCaP cell line with invasive phenotype

**Fig. 4:**
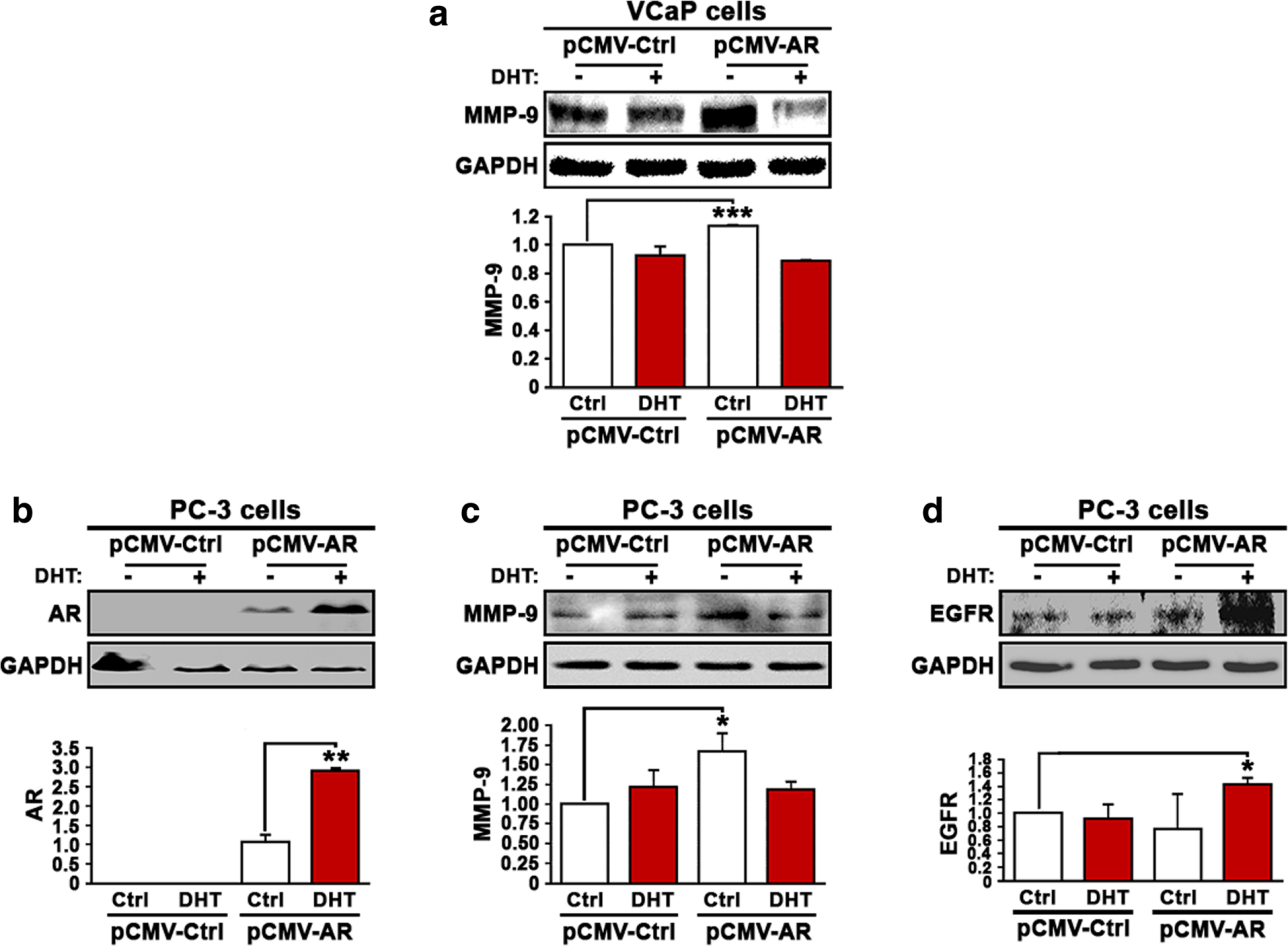
Evaluation of the effect of overexpression of AR in the presence or absence of DHT treatment on MMP-9 and EGFR expression in PCa cells. **a** Immunoblot analysis was performed to examine the expression of MMP-9 in VCaP cells that were transfected with pCMV control or pCMV-AR vectors followed by treatment with DHT or vehicle control. **b** Immunoblot analysis on the expression of AR in PC-3 cells that were transfected with pCMV control or pCMV-AR vectors followed by treatment with DHT. **c** Immunoblot analysis on the expression of MMP-9 in PC-3 cells that were transfected with pCMV control or pCMV-AR vectors followed by treatment with DHT. **d** Immunoblot analysis on the expression of EGFR in PC-3 cells that were transfected with pCMV control or pCMV-AR vectors followed by treatment with DHT. Data presented is the average of at least two independent experiments (±SD). *p* < 0.05 is indicated by “*”, *p* ≤ 0.01 is indicated by “**”, *p* ≤ 0.001 is indicated by “***”

MMP-9 is a key player in promoting metastatic dissemination and growth of PCa. To further elucidate the functional interlink between AR/EGFR and invasive signaling, we decided to analyze the relationship between AR, MMP-9 and EGFR signaling in PCa cell lines. We first examined whether DHT stimulation and AR overexpression may have any effect on MMP-9 expression in PCa cells. Interestingly, induced overexpression of AR in VCaP cells resulted in a significant increase in MMP-9 expression as compared with the control (*p* = 0.001) (Fig. 4a). However, combined DHT stimulation and AR overexpression did not increase MMP-9 expression (Fig. 4a). Thus AR, in the absence of its ligand androgen, is capable of inducing MMP-9 expression in VCaP cells.

To investigate whether there is a direct link between AR and MMP-9, we employed castration-resistant PC-3 cells, which lack endogenous AR expression. We introduced AR re-expression in PC-3 cells by transfecting the cells with pCMV-AR vector or pCMV control vector, followed by treatment of the transfected cells with DHT at 5 nM. AR expression was successfully induced in PC-3 cells, and DHT treatment further significantly increased AR expression (*p =* 0.005) (Fig. 4b). Similar to what was observed in VCaP cells, induced AR expression in PC-3 cells resulted in a significant increase in MMP-9 expression as compared with the control (*p* = 0.05) (Fig. 4c). However, combined DHT stimulation and AR overexpression did not further increase MMP-9 expression in PC-3 cells (Fig. 4c). These data suggest a direct link between AR and MMP-9 expression occurring independently of androgen.

We next examined EGFR expression in PC-3 cells expressing pCMV control vector or pCMV-AR vector in the absence or presence of DHT at 5 nM concentration. DHT alone showed no effect on EGFR expression in the absence of AR (Fig. 4d). Induced expression of AR alone had no effect on EGFR expression (Fig. 4d). Similar to what was observed in VCaP cells, combined AR expression and DHT treatment resulted in a remarkable increase in EGFR expression (*p* = 0.03) (Fig. 4d). Taken together, these results provide evidence suggesting that there is a positive and direct association between AR pathways and EGFR, and this signaling cascade is independent of stimulation or binding of AR by its ligand androgen.

**Fig. 5:**
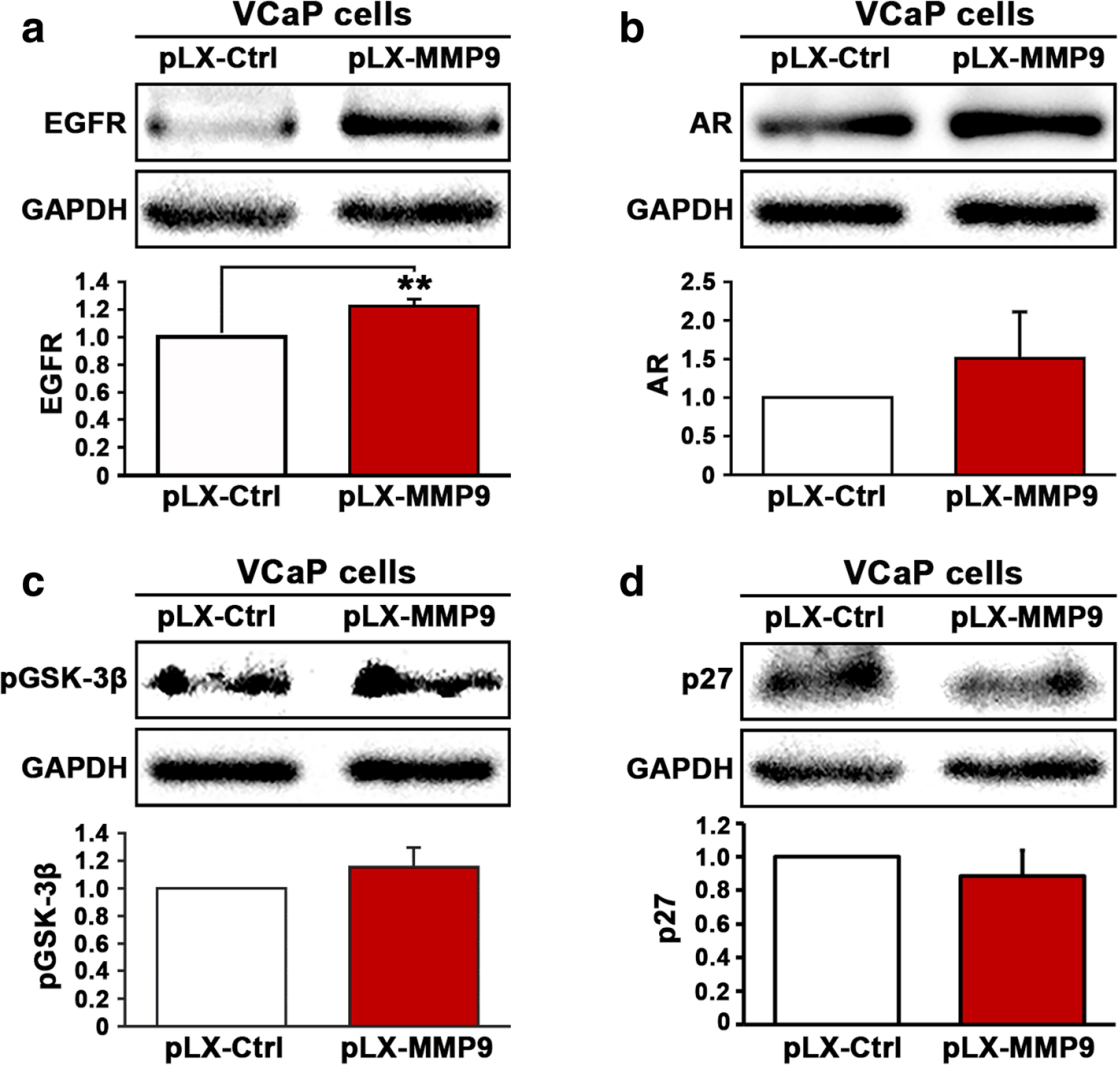
The effect of overexpression of MMP9 on the expression of EGFR, AR, p-GSK-3β and p27 in VCaP cells. **a** Immunoblot analysis was performed to examine the expression of EGFR in VCaP cells that were transfected with pLX-control vector (PLX-Ctrl) or pLX-MMP9 vector (PLX-MMP9). **b** Expression of AR in VCaP cells that were transfected with pLX-control vector (PLX-Ctrl) or pLX-MMP9 vector (PLX-MMP9). **c** and **d** Expression of p-GSK-3β and p27 in VCaP cells that were transfected with pLX-control vector (PLX-Ctrl) or pLX-MMP9 vector (PLX-MMP9). Data presented is average of two independent experiments (±SD). *p* < 0.05 is indicated by “*”, *p* ≤ 0.01 is indicated by “**”

Having demonstrated that enhanced AR signaling leads to increased expression of EGFR and MMP-9, we next wanted to investigate whether there might be a functional link between MMP-9 and EGFR. To this end, we induced overexpression of MMP-9 by transfecting VCaP cells with pLX-MMP-9 or pLX control vector. We found that induced overexpression of MMP-9 in VCaP cells led to a significant increase in EGFR expression (*p* = 0.01) (Fig. 5a). We also examined whether elevated expression of MMP-9 may have any effect on AR in VCaP cells. However, overexpression of MMP-9 had no significant effect on AR expression in VCaP cells (Fig. 5b). Overexpression of MMP-9 did not show significant effect on expression of the downstream targets of AKT including p-GSK-3β and p27 (Fig. 5c and d). Taken together, our results suggest that AR, EGFR and MMP9 are functionally interconnected in PCa cells.

**Fig. 6:**
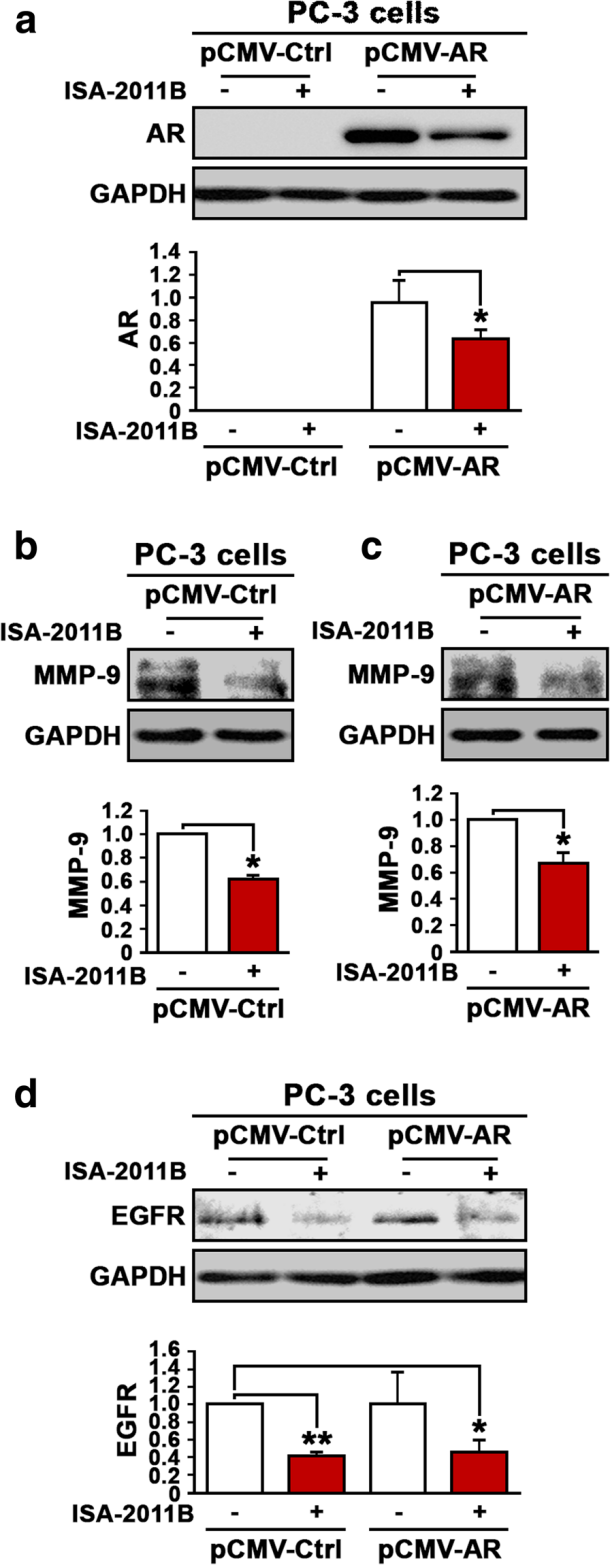
The effect of inhibition of the PI3K/AKT/AR axis on the expression of AR, MMP-9 and EGFR in PC-3 cells. **a** Immunoblot analysis on the expression of AR in PC-3 cells that were transfected with pCMV control or pCMV-AR vectors followed by treatment with PIP5K1α/AKT inhibitor ISA-2011B. **b** Immunoblot analysis on the expression of MMP-9 in PC-3 cells that were transfected with pCMV control vector followed by treatment with ISA-2011B. **c** Immunoblot analysis on the expression of MMP-9 in PC-3 cells that were transfected with pCMV-AR vector followed by treatment with ISA-2011B. **d** Immunoblot analysis on the expression of EGFR in PC-3 cells that were transfected with pCMV control or pCMV-AR vectors followed by treatment with ISA-2011B. Data presented is the average of at least two independent experiments (±SD). *p* < 0.05 is indicated by “*”, *p* ≤ 0.01 is indicated by “**”, *p* ≤ 0.001 is indicated by “***”

Next, we investigated whether inhibition of PI3K/AKT axis, the upstream regulator of AR signaling may have any effect on MMP-9 and EGFR expression in PCa cells. We employed PC-3 cells expressing control pCMV or pCMV-AR vectors, which previously provided a model system to examine the direct link between AR, MMP-9 and EGFR. We treated PC-3 cells that expressed pCMV control vector or pCMV-AR vector with ISA-2011B and examined the effect of ISA-2011B on AR expression. ISA-2011B treatment significantly reduced AR expression (*p =* 0.05) (Fig. 6a). Next, we examined the effect of ISA-2011B on MMP-9 expression in the absence or presence of AR expression in PC-3 cells. Interestingly, ISA-2011B treatment resulted in a significant down-regulation of MMP-9 in the absence of AR expression (*p =* 0.02) (Fig. 6b). ISA-2011B also significantly decreased MMP-9 expression in PC-3 cells expressing AR (*p =* 0.03) (Fig. 6c). Thus, MMP-9 expression can be inhibited by PIP5K1α inhibitor acting upstream the PI3K/AKT axis in the presence or absence of AR expression. Similar to what was observed in case of MMP-9, ISA-2011B treatment resulted in significant downregulation of EGFR expression in PC-3 cells in the absence or presence of AR expression (For EGFR in the absence of AR, *p =* 0.003, for EGFR in the presence of AR, *p =* 0.03) (Fig. 6d). This data further reinforces the hypothesis that the PI3K/AKT axis plays a fundamental role in mediating signaling between EGFR and AR in CRPC.

## Discussion

Under the castration-resistant state, despite the minimal levels of androgens, PCa cells are capable of growing rapidly and obtaining survival and invasive advantages (Semenas et al. 2012). AR is a transcriptional factor, which regulates a panel of genes controlling the growth of prostate cells. However, whether AR may be functionally linked to the EGFR and MMP-9 invasion pathways in the presence or absence of its ligand androgen remains poorly understood.

In this study, we investigated the clinical importance and link between AR, EGFR and MMP-9 in prostate cancer by using clinical tissues from prostate cancer patients and prostate cancer cell lines. One of our important new findings revealed that EGFR expression was elevated in metastatic PCa tissues. PCa patients with altered levels of EGFR mRNA expression in their primary or metastatic tumors suffered poorer DFS compared to those without alterations in EGFR expression. This suggests that elevated level of EGFR expression is associated with poor patient outcome in PCa patients. It is possible to hypothesize that EGFR protein up-regulation in advanced PCa may have either occurred from alterations at transcriptional level or alterations at post-translational level. Increasing evidence suggests that AR cross-talks with the EGFR axis and renders PCa cells independent of androgen (Brizzolara et al. 2017; Craft et al. 1999; Jathal et al. 2016; Pignon et al. 2009). In the present study, we investigated the association and interplay between AR and EGFR in PCa progression. We also found that there was a significant correlation between AR and EGFR mRNA expression in a large patient cohort obtained from public dataset. Further, there was a significant correlation between AR and EGFR protein expression in the patient cohort collected by our laboratory.

We found that DHT stimulation and AR overexpression significantly increased the level of EGFR in VCaP cells. Furthermore, simultaneous DHT treatment and AR overexpression increased the level of EGFR somewhat more pronouncedly than DHT treatment alone. These data shows that EGFR expression may be regulated by AR upon stimulation of androgen. We further showed that AR overexpression alone had no significant effect on p-GSK-3β or p27, however, DHT treatment and AR overexpression additively induced significant up-regulation of p-GSK-3β and significant down-regulation of p27 in VCaP cells. These data suggest that constitutive activation of elevated AR through its ligand DHT may further activate pathways downstream of EGFR including PI3K/AKT pathways, thus presumably allowing PCa cells to gain survival and invasive advantages. It has been revealed that EGFR-mediated activation of AKT occurs in part through dimerization of EGFR with HER3 or alternatively, through enhanced HER3 activity and in part via interaction of EGFR with the intracellular adaptor protein (Craft et al. 1999; Di Lorenzo et al. 2002; Turke et al. 2012). Simultaneous occurrence of EGFR and phosphatase and tensin homolog (PTEN) alterations as well as an interplay between these two factors can be observed in various cancers such as cancers of the brain, lung and prostate (Bratland et al. 2009; Chott et al. 1999; Wozniak et al. 2017). Our data provides evidence suggesting that AR is functionally linked to EGFR and its associated AKT pathways. EGFR and its ligands may enhance phosphorylation of AR or act as AR co-regulators to promote activation of its downstream genes in the presence of androgen. Our findings suggest that PCa with elevated expression of AR and EGFR may have increased survival and invasive ability of PCa cells.

MMP-9 is one of the key factors, which promote cancer metastasis and it is also a transcriptional target of AR, commonly present in metastatic PCa (Hu et al. 2016; Semenas et al. 2014). In the present study, we showed that induced AR expression increased MMP-9 expression in VCaP in the absence of DHT. To further investigate whether there is a direct association between AR and MMP-9, we used PC-3 cells, which lack endogenous AR expression. Induced AR expression led to a significant increase in MMP-9 expression in PC-3 cells in the absence of DHT treatment. These results suggest that there is a direct link between AR and MMP-9 in PCa cells, and that AR acts on MMP-9 independently of androgen.

In the present study, we showed that MMP-9 overexpression significantly increased EGFR expression in VCaP cells. Our finding that EGFR is up-regulated in MMP-9 overexpressing cells further reinforces the relationship between EGFR and AR signaling and the involvement of EGFR in invasion promoting signaling networks. MMP-9 as an extracellular matrix factors may be served as ligand to bind to and enhance EGFR protein stability. Alternatively, as shown in the reported studies, MMP9 enhance EGFR expression via PI3K/AKT pathways in cancers of the lung, ovaries, breast and brain (Chen et al. 2016; Comamala et al. 2011; Elbaz et al. 2015; Garrido et al. 2017; Pei et al. 2014). This hypothesis is further supported by the previous published studies suggesting that EGFR cascades of pathways may be associated with MMP-9 during dissemination of PCa cells PCa (Lue et al. 2011; Xiao et al. 2012; Zhu et al. 2013). Our results suggest that upon ligand stimulation, AR increases EGFR expression, which in turn acts on AKT pathways to promote cancer cell survival and invasiveness. In parallel, elevated level of AR increased MMP-9 expression, which also positively stimulated EGFR at an androgen-independent fashion. Our data provides new information suggesting that AR, EGFR and MMP-9 are interconnected and may play important roles during cancer progression from androgen-dependent state to castration-resistant state.

We investigated whether inhibition of PI3K/AKT axis, the upstream of AR signaling may have any effect on MMP9 and EGFR expression in PCa cells. ISA-2011B treatment significantly reduced AR expression. Next, we examined the effect of ISA-2011B on MMP9 expression in the absence or presence of AR expression in PC-3 cells. Interestingly, ISA-2011B treatment resulted in a significant down-regulation of MMP9 in the presence and absence of AR expression. This suggests that MMP-9 expression is influenced not only by AR signaling, but also by PI3K/AKT pathways. Thus, elevated level of MMP-9 may be inhibited by blocking PIP5K1α/PI3K/AKT survival pathways, which is in part related to AR in PCa cells. Similar to what was observed for MMP9, ISA-2011B treatment resulted in significantly down regulation of EGFR expression in PC-3 cells in the absence or presence of AR expression. Our data further provided new information on that elevated level of EGFR may be inhibited by blocking both PIP5K1α/PI3K/AKT and AR-androgen pathways in subsets of PCa patients with elevated levels of AR and EGFR in their tumors.

## Conclusions

In conclusion, our study provides an insight into the potential role of EGFR in advanced and invasive PCa possibly by acting as an upstream regulator of AR via the PI3K/AKT axis in growth and survival while likely acting through distinct pathways in invasive mechanisms. The study also provides a clue about the communication between the EGFR/AR axis and MMP-9, which might be a crucial component of tumor dissemination and establishment at the metastatic sites.

## Abbreviations

AR: Androgen receptor
BCR: Biochemical recurrence
CNA: Copy-number alteration
CRPC: Castration-resistant prostate cancer
CTCs: Circulating tumor cells
DFS: Disease-free survival
DHT: Dihydrotestosterone
ECM: Extracellular matrix
EGFR: Epidermal growth factor receptor
ER: Estrogen receptor
FBS: Fetal bovine serum
HER-2: Human epidermal growth factor receptor-2
IGF: Insulin-like growth factor
MMP-9: Matrix metalloproteinase-9
PCa: Prostate cancer
p-GSK-3β: Phospho-Glycogen synthase kinase-3-beta
PIP5K1α: Phosphatidylinositol 4-phosphate 5-kinase type-1 alpha
PSA: Prostate-specific antigen
PSN: Penicillin-streptomycin-neomycin
PTEN: Phosphatase and tensin homolog
TICs: Tumor initiating cells
TMAs: Tissue microarrays
VEGF: Vascular endothelial growth factor
